# Epidermal Growth Factor-Mediated Mitogen-Activated Protein Kinase3/1 Pathway Is Conducive to *In Vitro* Maturation of Sheep Oocytes

**DOI:** 10.1371/journal.pone.0120418

**Published:** 2015-03-23

**Authors:** Hemin Ni, Xihui Sheng, Xu Cui, Meichao Gu, Yunhai Liu, Xiaolong Qi, Shuhan Xing, Yong Guo

**Affiliations:** Animal Science and Technology College, Beijing University of Agriculture, Beijing, China; China Agricultural University, CHINA

## Abstract

Epidermal growth factor (EGF) has been shown to facilitate the *in vitro* maturation of sheep oocytes, and enhance embryo’s capability for further development. However, such kind of molecular mechanism has not yet been elucidated. In the present study, we investigated the effect of EGF-mediated mitogen-activated protein kinases 3 and 1 (MAPK3/1) pathway on *in vitro* maturation of sheep oocytes. U0126, a specific inhibitor of MEK (MAPK kinase), was added into the maturation culture medium to block the EGF-mediated MAPK3/1 pathway with different doses. Then, the nuclear maturation of sheep oocytes was examined. Additionally, the effect of EGF-mediated MAPK3/1 on cytoplasmic maturation was examined though *in vitro* fertilization and embryonic development. The rate of germinal vesicle breakdown (GVBD) after 6 h of culture with 10^−4^ mol/l of U0126 (50.4%) was significantly decreased compared with control (67.2%, *p* < 0.05), and the first polation body (PB1) extrusion rate after 22 h of culture in drug treatment was also significantly inhibited compared with control (28.6% vs. 48.4%, p < 0.05). However, 10^−6^ mol/l U0126 had slight effect on oocyte nuclear maturation. The normal distribution rate of α-tubulin in the oocytes after 22 h of *in vitro* maturation was significantly decreased in the 10^−4^ mol/l U0126 group (54%) compared with control (68%, *p* < 0.05). After *in vitro* fertilization, the cleavage rate in drug treatments (56.8% in 10^−6^ mol/l U0126 group and 42.6% in 10^−4^ mol/l U0126 group) was significantly decreased compared with control (72.3%, *p* < 0.01). The blastocyst rate in 10^−4^ mol/l U0126 group (17.6%) was also significantly decreased compared with control (29.9%, *p* < 0.05). Collectively, these results suggest that EGF-mediated MAPK3/1 pathway is conducive to *in vitro* maturation of sheep oocytes.

## Introduction

In mammals, luteinizing hormone (LH) from the pituitary induces a sequential and transient expression of the epidermal growth factor (EGF)-like growth factors such as epiregulin, amphiregulin, and betacellulin expressed in mural granulosa cells (MGCs) [1, 2]. These growth factors then activate common EGF receptor (EGFR) in cumulus cells to stimulate oocyte maturation [1, 3]. Recently, it has been reported that EGF-like growth factors could also regulate maternal mRNA translation and developmental competence of mouse oocytes by activation of the PI(3)K-AKT-mTOR pathway [4].

The oocyte is maintained in meiotic prophase arrest by natriuretic peptide type C (NPPC) acting via its cognate receptor, natriuretic peptide receptor 2 (NPR2) [5]. Some evidences show that EGFR signaling induces meiotic resumption by downregulating *Nppc* mRNA expression [6], and decreases NPR2 guanylyl cyclase activity via the elevation of calcium concentrations of cumulus cells [7]. Both of which lead to the decrease of cGMP levels in the follicle [8–10]. Anyway, the key downstream effectors of EGFR signaling in cumulus cells, mitogen-activated protein kinases 3 and 1 (MAPK3/1, also known as ERK1/2), is essential for mammalian oocyte maturation [3, 10, 11]. It has been demonstrated that, in mouse and pig oocytes, MAPK3/1 pathway is essential for spindle assembly and microtubule organization during mammalian oocyte meiosis [12, 13]. In bovine oocytes, it is responsible for MII arrest, maintenance of maturation-promoting factor (MPF) activity, and spindle organization [14].

The maturation of mammalian oocytes is a complex and dynamic process involving the maturation of nucleus and cytoplasm [15]. Only oocytes with a mature nucleus and cytoplasm are able to support normal fertilization and further embryonic development [16, 17]. At present, the acquirement of large livestock’s mature oocytes is difficult, so it is necessary to obtain massive *in vitro* matured oocytes with high quality, in spite that the developmental competence of oocytes matured *in vitro* is markedly inferior to that of their *in vivo* matured counterparts [18–20].

Many studies show that the activation of EGFR by the addition of EGF *in vitro* can facilitate the maturation of sheep oocytes, and enhance the embryo’s capability for further development [21–23]. However, the mechanism is unclear. This study was aimed to investigate the effect of EGF-mediated MAPK3/1 pathway on *in vitro* maturation of sheep oocytes. U0126, a specific inhibitor of MEK (MAPK kinase), was added to the maturation culture medium to block the EGF-mediated downstream MAPK3/1 pathway. Then, the maturation of nucleus was examined. Additionally, the cytoplasmic maturation was examined through *in vitro* fertilization and embryonic development.

## Materials and Methods

### Ethics Statement

Animal welfare and experimental procedures were carried out in accordance with the Guide for the Care and Use of Laboratory Animals (Ministry of Science and Technology of China, 2006), and were approved by the animal ethics committee of Beijing University of Agriculture.

### Oocyte collection and maturation culture

Sheep ovaries were collected from Fuhua slaughterhouse (Dachang Hui Autonomy County, Hebei province) and were immediately placed in 25–28°C normal saline containing penicillin-streptomycin, then transported to the laboratory within 2–4 h. The ovaries were washed three times in normal saline and trimmed to remove fat and the corpus luteum. Then, ovaries were placed in a petri dish containing oocyte pick-up solution (Table 1) and removed the ovarian follicle. According to the test needs, cumulus-oocyte complexes were sorted out under a stereomicroscope (Olympus SZ40, Tokyo, Japan). The selection criteria were as follows: complete morphology, dense cytoplasm, uniform color, at least three layers of granular cells, and dense encapsulation.

**Table 1:** The recipes of reagents.

Reagent	Recipe
Oocyte pick-up solution	TCM199 + 25 mmol/l HEPES + 2.2 mg/ml NaHCO_3_ + 2% FCS + 100 IU/ml streptomycin + 100 μg/ml penicillin
Maturation culture medium I	TCM199 + 10 mmol/l HEPES + 2.2 mg/ml NaHCO_3_ + 8 mg/ml BSA + 0.25 mmol/l sodium pyruvate + 2.75 mmol/l lactate + 100 IU/ml penicillin + 100 μg/ml streptomycin + 50 ng/ml EGF +1 μg/ml E_2_ + 10 μg/ml LH + 10 μg/ml FSH
Maturation culture medium II	Maturation culture medium I with 10^−6^ mol/l U0126
Maturation culture medium III	Maturation culture medium I with 10^−4^ mol/l U0126
SOF working solution	SOF stock solution + 1 mmol/l glutamine + 0.3 mmol/l sodium pyruvate
Oocyte washing solution	SOF working solution + 10 mmol/l HEPES + 5 mmol/l NaHCO_3_ + 0.3% BSA + 100 IU/ml penicillin + 100 μg/ml streptomycin
Sperm-washing solution	The same ingredients as oocyte washing solution, with a double amount of double antibody
Capacitation solution	SOF working solution + 20% estrous sheep serum + 10 mmol/l penicillamine + 10 mmol/l hypotaurine + 10 μg/ml heparin + 0.5 mol/l calcium lactate + 100 IU/ml penicillin + 100 μg/ml streptomycin
Embryo culture medium	SOF working solution + 10% FCS + 2% essential amino acids (BME-EAA) + 1% nonessential amino acids (MEM-NEAA) + 100 IU/ml penicillin + 100 μg/ml streptomycin

U0126 (Sigma-Aldrich, MO, USA) was added into the maturation culture medium with different concentrations (0, 10^−6^, and 10^−4^ mol/l), and each group had three replicates. Then three groups of oocytes were respectively rinsed using different maturation culture medium by two times and transferred into 50 μl of pre-equilibrated maturation medium droplets (10 oocytes each). The incubation was performed at 38.5°C in an atmosphere of 5% CO_2_ under saturated humidity.

### Determination of oocyte nuclear maturation

#### Examination of GVBD.

Examination of GVBD. After 6 h of *in vitro* maturation, different groups of oocytes were treated with the oocyte washing solution containing 0.1% hyaluronidase by mechanical pipetting to remove cumulus cells. The obtained oocytes were placed on a glass slide with droplets of paraffin wax: vaseline (1:9, v/v) in the four corners. Cells were covered with a coverslip and then fixed for more than 24 h in ethanol: acetic acid (3:1, v/v). Then, cells were stained with 1% aceto-orcein for 1–2 min. GVBD were examined under a biological microscope (Nicon YS2, Tokyo, Japan).

#### Examination of PB1 extrusion.

After 24 h of *in vitro* maturation, different groups of oocytes were subjected to mechanical pipetting with the oocyte washing solution containing 0.1% hyaluronidase to remove cumulus cells. The extrusion of PB1 was examined under a stereomicroscope (Olympus SZ40, Tokyo, Japan) and taken as an indication of oocyte nuclear maturation for data analysis.

#### Immunofluorescence labeling of α-tubulin.

Different experimental groups of sheep oocytes were collected at varying maturity periods (4, 8, 12, and 24 h). The oocytes were digested in 0.5% hyaluronidase to completely remove granulosa cells, and the zona pellucida was removed with phosphate-buffered saline (PBS) (pH 2.5). The digested oocytes were fixed in 4% paraformaldehyde at room temperature for 20 min and then placed in 0.2% Triton-X100 (Sigma-Aldrich, MO, USA) for 30 min of osmosis. Thereafter, oocytes were incubated in a blocking agent (PBS +2% BSA + 10% goat serum + 2% skim milk powder + 0.15 mol/l glycine) at 37°C for 1 h, followed by the addition of FITC-conjugated mouse anti-human α-tubulin monoclonal antibody (ab64503) (Abcam DM1A, Cambridge, UK) with a final concentration of 1 μg/ml [24] and incubation at 37°C for another 1 h. The immunogen of antibody is full length native protein (purified) of Chicken alpha Tubulin (extracted from brain). At the end of incubation, oocytes were thoroughly washed in 0.2% Triton-X100, 5 μg/ml propidium iodide (Sigma-Aldrich, MO, USA) was added, and then they were placed in a cassette for 10 min of nuclide labeling. Finally, oocytes were examined under a confocal microscope (ZEISS LSM 510 META, Oberkochen, Germany).

### *In vitro* fertilization of oocytes and embryo culture

#### Sperm capacitation.

Fresh semen was washed twice with sperm-washing solution by centrifugation at 1500 rpm/min for 5 min. The supernatant was decanted and sperm at the bottom of the centrifuge tube was added to the pre-equilibrated capacitation solution for 30 min at 38.5°C in an atmosphere of 5% CO_2_ under saturated humidity.

#### *In vitro* fertilization.

Mature oocytes were digested with 0.5% hyaluronidase to partially remove granulosa cells. The oocytes were then washed thrice with the capacitation solution and transferred into fertilization droplets. Sperm was added to the fertilization droplet containing oocytes. Each drop contained 10 μl of sperm with a density of 5 × 10^6^ sperm/ml. The oocyte-sperm complex was incubated for 17–19 h at 38.5°C in a 5% CO_2_ atmosphere under saturated humidity.

#### Embryo culture.

After 17–19 h of oocyte-sperm co-incubation, zygotes were washed thrice with the oocyte washing solution to remove granulosa cells and sperm. After twice washes with the embryo culture medium, zygotes were transferred into droplet containing monolayer granulosa cell and incubated at 38.5°C in a 5% CO_2_ atmosphere under saturated humidity. Half the medium was exchanged with new medium every other day. The cleavage rate was determined at 48 h and the blastocyst rate was estimated at 7d.

### Data analysis

All experiments were performed at least 3 times. The data were subjected to chi-square analysis using SAS 9.0 statistical software (SAS Institute Inc., Cary, NC, USA). A *p-*value less than 0.05 was considered statistically significant.

## Results

### Oocyte nuclear maturation in different treatment groups

The GVBD rate of sheep oocytes was examined after 6 h of *in vitro* maturation. After 6 h of *in vitro* maturation, the GVBD rate of mature sheep oocytes in the 10^−4^ mol/l U0126 group (50.4%) was significantly decreased compared with control (67.2%, *p* < 0.05). There was no significant differenceS between the 10^−6^ mol/l U0126 group and the control group (55.2% vs 67.2%, *p >* 0.05) (Table 2).

**Table 2:** Statistics of germinal vesicle breakdown (GVBD) rate of sheep oocytes in different treatment groups after 6 h of *in vitro* maturation.

Group	Total number of oocytes	Number of GVBD oocytes	GVBD rate (%)
Control	113	76	67.2 (76/113)^a^
10^−6^ mol/l U0126 group	105	58	55.2 (58/105)^ab^
10^−4^ mol/l U0126 group	105	53	50.4 (53/105)^b^

Note: The same superscript letters in the same column indicate no statistically significant differences (*p* > 0.05); different superscript letters in the same column indicate statistically significant differences (*p* < 0.05).

**Fig 1:**
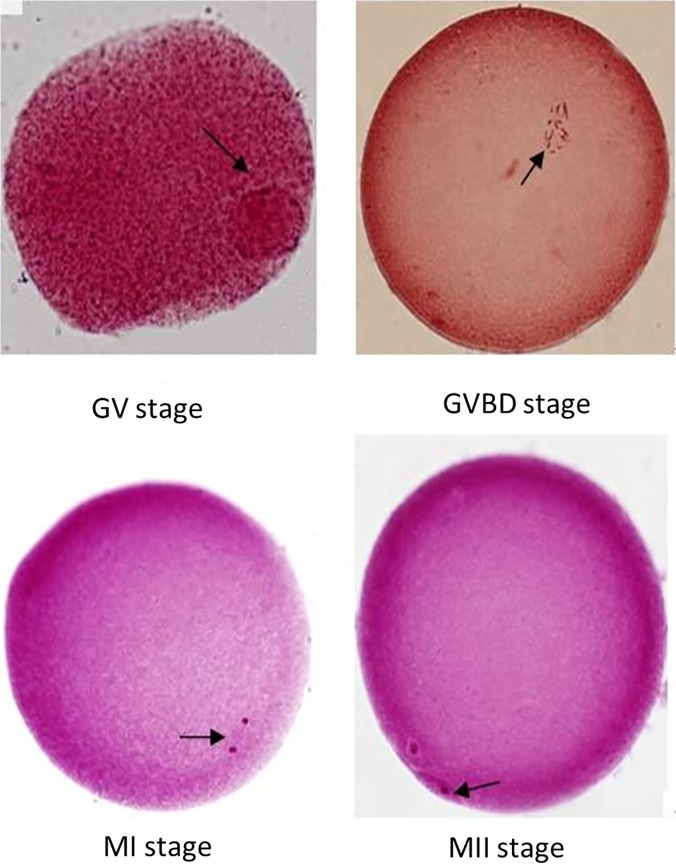
Microscopic characteristics of sheep oocyte maturation stained with aceto-orcein (200× magnification). (A) Oocyte at GV stage, arrow points to germinal vesicle; (B) oocyte at GVBD stage, arrow points to condensed chromosomes; (C) oocyte at late spindle stage, arrow points to spindle fibers; and (D) oocyte at middle MII stage, arrow points to the first polar body.

The PB1 extrusion rate of sheep oocytes was examined after 24 h of *in vitro* maturation. After 24 h of culture, the PB1 extrusion rate of mature sheep oocytes in the 10^−4^ mol/l U0126 group (28.6%) was significantly decreased compared with control (48.4%, *p* < 0.05). There was no significant differences between the 10^−6^ mol/l U0126 group and the control group (32.3% vs 48.4%, *p >* 0.05) (Table 3). Microscopic characteristics of sheep oocyte maturation stained with aceto-orcein are illustrated in Fig. 1.

**Table 3:** Statistics of the first polar body (PB1) extrusion rate of sheep oocytes in different treatment groups after 24 h of *in vitro* maturation.

Group	Total number of oocytes	Number of oocytes with PB1 extrusion	PB1 extrusion rate(%)
Control	62	30	48.4(30/42)^a^
10^−6^ mol/l U0126 group	65	21	32.3(21/65)^ab^
10^−4^ mol/l U0126 group	70	20	28.6(20/70)^b^

Note: The same superscript letters in the same column indicate no statistically significant differences (*p* > 0.05); different superscript letters in the same column indicate statistically significant differences (*p* < 0.05).

### The distribution of α-tubulin in different treatment groups

**Fig 2:**
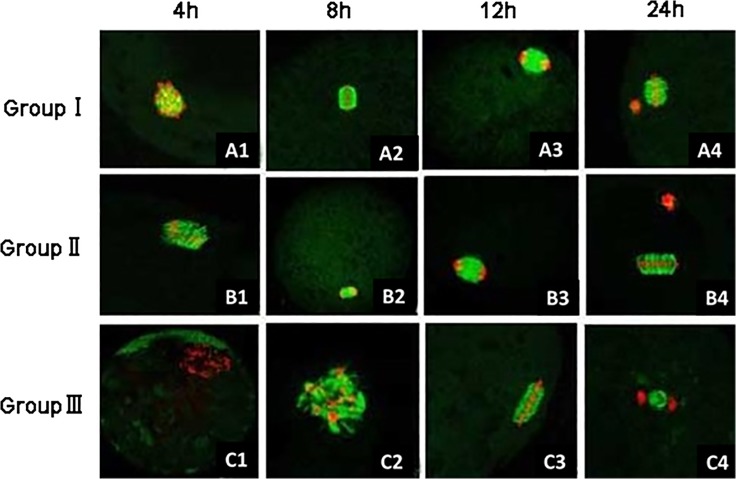
The α-tubulin distribution around chromosomes in sheep oocytes after different periods of *in vitro* maturation. Red indicates chromosomes and green indicates α-tubulin. (A1-C1, 4 h) Oocytes show GVBD in the control group (group I, A1, 10×40) and the 10^−4^ mol/l U0126 group (group III, C1, 10×20), but remain in the germinal vesicle stage in the 10^−6^ mol/l U0126 group (group II, B1,10×40); (A2-C2, 8 h) chromosome condensation (A2, 10×40), beginning of chromosome condensation (B2,10×20), and GVBD (C2, 10×40); (A3-C3, 12 h) telophase of meiosis I (A3, 10×20; B3, 10×40), and chromosome condensation (C3, 10×40); (A4–C4, 24 h) meiosis II (A4, B4, 10×40), and telophase of meiosis I (C4, 10×20).

The distribution of α-tubulin in sheep oocytes at different maturity periods were examined by immunofluorescence staining (Fig. 2). As the culture time elapsed, the amount of α-tubulin distributed around chromosomes increased. In treatments, the occurrence of α-tubulin aggregation around chromosomes and the spindle formation in oocytes were later than that in control. After 18 h of *in vitro* maturation, 69% (48/70) of oocytes in the control group entered anaphase of meiosis, some oocytes even entered the metaphase of meiosis, i.e., the MII stage. But in the 10^−4^ mol/l U0126 group, approximately 50% (36/71) of oocytes remained in the metaphase of meiosis I.

### The distribution of α-tubulin in sheep oocyte nucleus after 22 h of in vitro maturation

**Fig 3:**
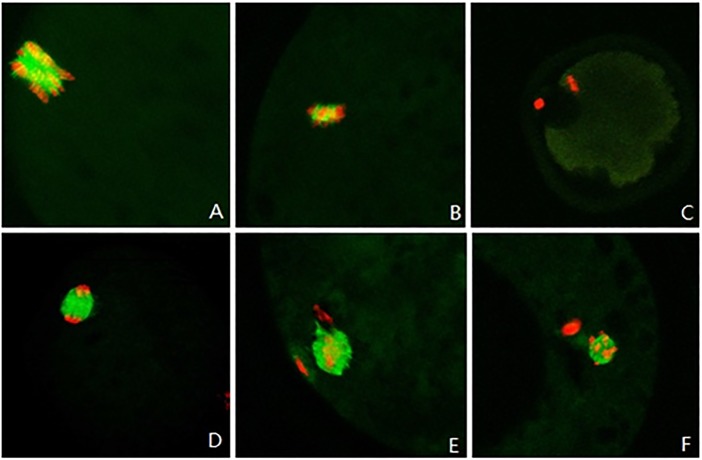
The α-tubulin distribution around chromosomes in sheep oocytes after 22 h of *in vitro* maturation. Red indicates chromosomes and green indicates α-tubulin. (A, 10×40) Disorderly distribution of chromosomes; (B, 10×40) little α-tubulin distributed around chromosomes; (C,10×20) nearly no α-tubulin distributed around chromosomes; (D, 10×40) α-tubulin distributed on a spindle; and (E, F, 10×40) formation of microtubules and extrusion of polar bodies at meiosis II.

The experimental results were interpreted in accordance with the classification of α-tubulin by Miyara [14]. Fig. 3A–C show an abnormal distribution of α-tubulin, and Fig. 3D–F show a normal distribution. Statistics of the distribution of α-tubulin in different groups of sheep oocytes after 22 h of *in vitro* maturation are shown in Table 4.

**Table 4:** Statistics of the distribution of α-tubulin of sheep oocytes in different treatment groups after 22 h of *in vitro* maturation.

Group	Total number of oocytes	Number of oocytes with normal distribution of α-tubulin	Normal distribution rate of α-tubulin (%)
Control	152	103	68a
10^−6^ mol/l U0126 group	149	88	59^ab^
10^−4^ mol/l U0126 group	155	85	54^b^

Note: The same superscript letters in the same column indicate no statistically significant differences (*p* > 0.05); different superscript letters in the same column indicate statistically significant differences (*p* < 0.05).

Statistical analysis showed that the normal distribution rate of α-tubulin in sheep oocytes after 22 h of *in vitro* maturation decreased with an increasing dose of the inhibitor, with 54% in the 10^−4^ mol/l U0126 group, significantly lower than 68% in the control group (*p* < 0.05). This indicated that the addition of U0126 affected normal expression of α-tubulin and its distribution in sheep oocytes.

### In vitro fertilization of sheep oocytes in different treatment groups

After *in vitro* fertilization, sheep oocytes in theatments had significantly lower cleavage rates than those in the control group (*p* < 0.01). The blastocyst rate of oocytes in the 10^−4^ mol/l U0126 group (13.0%) was significantly decreased compared with control (29.9%, *p <* 0.05), with no significant differences between the 10^−6^ mol/l U0126 group and the control group (24.6% vs 29.9%, *p >* 0.05) (Table 5).

**Table 5:** The cleavage and blastocyst rates of sheep oocytes in different treatment groups after *in vitro* fertilization.

Group	Number of embryos transferred	Number of cleaved embryos	Cleavage rate (%)	Number of blastocysts	Blastocyst rate (%)
Control	137	99	72.3 (99/137) ^A^	41	29.9(41/137)^a^
10^−6^ mol/l U0126 group	118	67	56.8 (67/118) ^Ba^	29	24.6(29/118)^a^
10^−4^ mol/l U0126 group	108	46	42.6 (46/108) ^Bb^	19	17.6(19/108)^b^

Note: The same superscript letters in the same column indicate no statistically significant differences (*p* > 0.05); different superscript letters in the same column indicate statistically significant differences (*p* < 0.05).

## Discussion

EGF has been shown to facilitate the *in vitro* maturation of sheep oocytes, and enhance embryo’s capability for further development [21–23]. However, its molecular mechanism underlying which has not been defined clearly. This study focused on investigating the effect of the EGF-mediated MAPK3/1 pathway on *in vitro* maturation of sheep oocytes. We used U0126, a specific inhibitor of MEK, to block the EGF-mediated MAPK3/1 pathway. The results showed that the addition of U0126 decreased the GVBD rate and the PB1 extrusion rate of sheep oocytes, and affected the normal expression and the distribution of α-tubulin in sheep oocytes. Further study showed that U0126 could reduce the cleavage and blastocyst rate after *in vitro* fertilization. Thus, these results suggest that EGF-mediated MAPK3/1 pathway is conducive to *in vitro* maturation of sheep oocytes.

As a specific inhibitor of MEK, U0126 has been widely used in studies of oocyte maturation with effective inhibition of MAPK3/1 activity [25–27]. More than 80% inhibition of MEK enzymic activity could be achieved by 10^−6^–10^−5^ mol/l U0126 in somatic cells [28]. In our study, 10^−6^ mol/l U0126 had no effect on the nuclear maturation and blastocyst development, but 10^−4^ mol/l U0126 exhibited a significant effect on them. These results were consistent with previous studies that block of ERK1/2 activity by 10^−5^ mol/l U0126 only slightly inhibited EGF-induced meiotic resumption [7], and only 10^−4^ mol/l U0126 could completely inhibit the effect of LH-induced GVB in cultured mouse follicles [29].

MAPK3/1 are co-expressed in all mammalian tissues and play a pivotal role in GVBD in oocytes. While the activation time of MAPK3/1 were variable among species. In rat, mouse and goat, their activation occurred after GVBD, which meant that they just involved in regulating post-GVBD events and were not required for GVBD [30–32]. In pig, cattle and Xenopus, MAPK3/1 were activated before GVBD or synchronously to induce GVBD [33–35]. However, the effect of MAPK3/1 on sheep GVBD was unknown. As can be seen from our results, addition of U0126 inhibited EGF-induced GVBD. This indicates that the MAPK3/1 pathway could affect the occurrence of GVBD in sheep.

The PB1 extrusion rate as a symbol of the oocyte nuclear maturation, is very important for embryo development after *in vitro* fertilization. Sakaguchi reported that the supplement of EGF and insulin-like growth factor-1 (IGF-1) could significantly increase the frequency of oocytes with PB1 at 16 h of culture (*p* < 0.05) [36]. But the mechanism is unclear. Our results showed that the PB1 extrusion rate of mature sheep oocytes in the 10^−4^ mol/l U0126 group was significantly decreased compared with control (28.6% vs 48.4%, *p* < 0.05), indicating that EGF could promote the extrusion of PB1 during sheep oocyte maturation *in vitro* via MAPK3/1 pathway.

In oocytes, after GVBD and PB1 extrusion, chromosomes in secondary oocytes form a metaphase plate with the long axis parallel to the cell membrane surface, which indicates that the oocyte nucleus has matured. The distribution of chromosomes and the morphology of the spindle in mature oocytes impact the development of the fertilized oocyte. Disorderly spindle assembly can cause abnormal distribution of chromosomes in oocytes, thereby preventing the extrusion of the first and the second polar body. It may also lead to the formation of aneuploid zygotes [37]. Therefore, the assembly and normal distribution of tubulin should be one of the focuses for evaluating the quality of the mature oocyte nucleus. Our results showed that, among different treatments of sheep oocytes, there existed obvious morphological differences of the aggregation and the distribution of α-tubulin around chromosomes after 22 h of *in vitro* maturation. The rate of oocytes in the telophase of meiosis in the control group was significantly increased compared with the drug treatments. Combined comparison with the distribution of α-tubulin at different time showed that the above phenomenon was possibly related to the speed of α-tubulin aggregation around chromosomes and the formation of microtubules, thereby affecting the distribution and separation time of chromosomes. These showed that U0126 significantly inhibited the expression of α-tubulin and its aggregation around chromosomes. This demonstrated that the EGF-mediated MAPK3/1 pathway could promote normal distribution of chromosomes and α-tubulin in sheep oocytes, further improving the quality of oocytes’ nuclear maturation, which were consistent with previous observations in mouse and pig oocytes [38, 39]. Furthermore, EGF has been shown to exert positive effects on cleavage rate and blastocyst formation during *in vitro* maturation process of sheep oocytes [21–23, 40]. Our results showed that MAPK3/1 affected the cleavage and blastocyst rates after *in vitro* fertilization of oocytes. All of these results demonstrate that EGF could regulate *in vitro* maturation of sheep oocytes via the MAPK3/1 pathway.

However, some studies suggest that MAPK3/1 are necessary but not sufficient to induce oocyte maturation. In cultured cumulus-oocyte complexes, activation of MAPK3/1 in cumulus cells with growth differentiation factor 9 (GDF9) alone is not sufficient to stimulate oocyte maturation [29]. Reduced but measureable levels of phosphorylated MAPK3/1 are induced by LH in *Areg* ^*−/−*^ *Egfr* ^*wa2/wa2*^ follicles, yet oocyte meiotic resumption is impaired [41]. Recent studies have demonstrated that an additional pathway may involved in LH-induced oocyte maturation. It has been shown that NPPC increases cGMP levels in granulosa cells via activation of NPR2, then the cGMP diffusing into oocyte via gap junctions, where it acts to maintain meiotic arrest by inhibiting phosphodiesterase (PDE) 3A activity and cAMP hydrolysis [2, 42, 43]. In addition, activation of LH receptors decreases both *Nppc* and *Npr2* mRNA expression [44, 45]. LH treatment also results in a reduction in NPR2 activity in mouse ovarian follicles, contributing to the decrease of cGMP leveles [45]. Thus, LH-induced decrease in NPPC content and NPR2 activity may reduce cGMP levels in the follicle, which enabling the oocytes to resume meiosis [45]. However, both MAPK3/1 pathway and NPPC/NPR2 are essential components of the LH signaling required to oocyte maturation, the correlation between them remains unclear. Further investigations into that will provide a better understanding of oocyte maturation in mammals and will be helpful for further improving the *in vitro* culture system of sheep oocytes.
